# Environmental and household factors associated with depressive symptoms in rural Kilifi County, Kenya: a machine learning analysis

**DOI:** 10.3389/fpubh.2026.1826112

**Published:** 2026-09-23

**Authors:** John Andrew, Ashley Njoroge, David Andai, Edna Bosire, Jasmit Shah

**Affiliations:** 1 Brain and Mind Institute, Aga Khan University, Nairobi, Kenya; 2 Department of Population Health, Aga Khan University, Nairobi, Kenya; 3 Department of Medicine, Aga Khan University, Nairobi, Kenya

**Keywords:** climate, depression, environmental factors, Kenya, Kilifi, machine learning, PHQ-9, SHAP

## Abstract

**Background:**

Depression is a leading global cause of disability, that disproportionately affects populations in low-resource settings in sub-Saharan Africa, where poverty, limited healthcare, and environmental stressors such as climate variability, poor water and sanitation, and household air pollution compound vulnerability. In Kilifi County, Kenya, recurrent droughts and economic insecurity amplify these challenges, yet the associations between environmental exposures and mental health impacts remain poorly characterized. This study applies machine learning models to examine how climate perceptions, water and sanitation access, and household air pollution proxies are associated with depressive symptoms in Kilifi County.

**Methodology:**

This cross-sectional study analyzed primary household survey data collected from July to December 2021 within the Kaloleni/Rabai Community Health and Demographic Surveillance System. Structured surveys captured demographics, mental health status via the Patient Health Questionnaire-9 (PHQ-9), and environmental exposures, including climate perceptions, agricultural stressors, water and sanitation access, and air pollution proxies. Depression was defined as PHQ-9 ≥ 10. Thirty-one variables were grouped into environmental perceptions, climate impacts, air pollution proxies, and water/sanitation categories. Random Forest and XGBoost models were trained in Python using 80/20 household-grouped train-test split (GroupShuffleSplit) to prevent data leakage, with 10-fold stratified group cross-validation. Class imbalance was addressed using balanced class weights (Random Forest) and scale_pos_weight (XGBoost). Model performance was assessed using accuracy, balanced accuracy, ROC-AUC, PR-AUC, sensitivity, specificity, precision, recall, F1-scores, and Brier scores with bootstrap 95% confidence intervals. Feature contributions were evaluated using Gini importance, gain and SHAP values.

**Results:**

Among 17,738 participants (median age 41.0 [IQR 31.0–54.0], 77.8% female), 14.9% (*n* = 2,635) screened positive for depression (PHQ-9 ≥ 10). XGBoost outperformed Random Forest across all metrics: 0.802 (95% CI: 0.788–0.815) versus 0.796 (95% CI: 0.782–0.808), ROC-AUC 0.845 (95% CI, 0.826–0.862) versus 0.823 (95% CI, 0.804–0.841), balanced accuracy 0.753 versus 0.738, and sensitivity 0.684 versus 0.657. Both models showed moderate precision for depression-positive cases (XGBoost 0.40, Random Forest 0.38) reflecting the precision-sensitivity trade-off inherent in class-imbalance handling. Key predictors across Gini importance, gain and SHAP analyses included drinking water source, heat-related health risks, drought exposure, home thermal discomfort and cooking with open fire highlighting the convergence of water/sanitation, climate, and household air quality factors.

**Conclusion:**

Environmental and household exposures are consistently associated with depression in rural Kilifi County. Machine learning identified water and sanitation conditions, climate variability perceptions, and household energy practices as leading predictors. Integrating environmental and climate-sensitive indicators into community health workflows, alongside interventions such as clean cookstoves, water access and climate-resilient agriculture, could reduce depression burden and enhance precision public health for climate-vulnerable communities. Longitudinal studies with refined exposure measurement are needed to clarify the temporal and causal nature of these associations.

## Introduction

Although the mental health impacts of climate change are less extensively studied in developing nations, growing evidence from developed countries indicates that environmental shifts can significantly influence psychological well-being. These effects occur through both direct and indirect mechanisms, particularly impacting individuals with pre-existing vulnerabilities or those residing in ecologically fragile regions (1). These impacts may include psychological distress, depression and anxiety, and increased addictions and suicide rates (2). For example, specific mental health sequelae of acute or more prolonged periods of heat exposure are considerable, including generalized anxiety and depression (2, 3). Air pollution can also cause serious neurocognitive effects ranging from behavioral variations to neurodegenerative disorders that can ultimately have devastating effects on mental health (4, 5). A growing body of research has linked air pollution, both at ambient and household levels, with mental health disorders such as depression, stress, anxiety, suicidal behavior, cognitive impairment, and dementia (6, 7). Although interventions aimed at addressing public health effects of climate change have been proposed and are being debated at global level (8, 9), none of these are specific to mental health and none target low and middle income countries (LMICs).

From an environmental psychology perspective, the relationship between environmental conditions and mental health operates through multiple pathways. Lazarus and Folkman’s transactional model of stress and coping (10) provides a useful framework: individuals appraise environmental stressors (such as drought, heat, or inadequate water access) in terms of their perceived threat to well-being and their available coping resources. When environmental demands exceed perceived coping capacity, chronic psychological distress may result. This is particularly relevant in resource-constrained settings where adaptive capacity is limited. The concept of “place-based stress” further suggests that degraded or threatening home and neighborhood environments can erode psychological resilience through persistent low-level stressors, loss of environmental control, and disruption of daily routines (11). Perceived environmental deterioration — including subjective assessments of climate change, thermal discomfort, and resource scarcity — may therefore serve as both indicators of objective environmental conditions and independent contributors to psychological burden through cognitive appraisal processes (12).

The exposome framework, which encompasses the totality of environmental exposures from conception onward (13), offers a complementary lens for understanding how multiple co-occurring household and environmental stressors may cluster to influence mental health. In rural sub-Saharan African settings, exposures such as indoor air pollution from biomass fuels, inadequate water and sanitation, heat stress, and climate-driven livelihood disruption frequently co-occur within the same households and communities, creating compound exposure profiles that traditional single-exposure analyses may fail to capture (14, 15).

The impacts of climate change in sub-Saharan Africa (SSA) are well documented (16–18). Populations in this region are vulnerable to extreme weather events, including heatwaves, tropical cyclones, torrential rains, flooding, wildfires, and droughts, which pose complex threats to both their physical safety and livelihoods (19). These factors are significant contributors to both mental and physical health challenges (14, 20). In 2019, air pollution was responsible for 1.1 million deaths across Africa in which indoor air pollution accounted for 697,000 deaths and ambient air pollution for 394,000 (15). A recent study from India has shown that exposure to indoor air pollutants, i.e., cooking fuel, tobacco smoke are risk factors for cognitive impairment especially among older adults (21); and another study from South Africa has shown an association of prenatal exposure to indoor air pollution (including tobacco smoke) on neurodevelopment at two years of age (22). Further, a recent systematic review conducted in low and middle income countries (LMICs) including Kenya has reported that lack of access to safe water and adequate sanitation has implications for the psychosocial well-being of individuals and households (23).

Understanding the association between climatic changes, household environmental conditions, and depressive symptoms in resource constrained settings in LMICs is important because the impacts of climate change are often unequal, disproportionately affecting marginalized populations in socio-economically deprived settings. In Kenya, extreme weather and climate events such as droughts, high temperatures, heavy rainfall and floods have been witnessed in many parts of the country with associated risks and impacts on human life (24–26). The Kenyan coastal region for example, is on low altitude close to sea level, and populations living in these contexts are often exposed to extremes of prolonged droughts and flooding which occur almost annually (24). In addition, heavy rainfall and storms have led to increased frequency of flooding and population displacement, destruction of property and infrastructure, negatively impacting access to water and sanitation, loss of biodiversity and food security (24). Residents of urban slums and rural regions are disproportionately impacted by these climate shocks, owing to their limited access to water, food, and healthcare services for both mental and physical health conditions (27).

Given that many of the environmental variables relevant to mental health in these settings are subjective or semi-subjective — including perceived changes in rainfall, thermal discomfort at home, and self-reported drought exposure — the integration of environmental psychology and cognitive appraisal frameworks is essential for interpreting their associations with depressive symptoms. Individuals experiencing depressive symptoms may perceive and report environmental conditions differently from those who are not depressed, a consideration that is particularly salient in cross-sectional designs (12).

Machine learning approaches are well suited to modeling these complex, potentially nonlinear associations across multiple correlated environmental domains. Unlike traditional regression methods, tree-based ensemble algorithms such as Random Forest and XGBoost can capture interactions among predictors without requiring explicit specification, and can accommodate mixed variable types and non-additive relationships (28, 29). This study had three objectives: (1) to characterize the prevalence and severity distribution of depressive symptoms; (2) to examine associations between self-reported environmental and household exposures and screening-positive depression using machine learning classification; and (3) to identify which environmental and household features contribute most to predictive models of depression screening status within the Kaloleni/Rabai Health and Demographic Surveillance System (KRHDSS) cohort in Kilifi, Kenya (30).

## Materials and methods

### Study design and setting

We conducted a cross-sectional analytical study utilizing primary household survey data collected between July and December 2021 from households distributed across multiple rural community health units (CHUs) in Kilifi County, Kenya as part of the Kaloleni/Rabai Community Health and Demographic Surveillance System. The surveillance system, established by the Aga Khan University (AKU) in collaboration with the Kaloleni and Rabai Sub-County Health Management Offices, is a population-wide demographic and health registry nested within the Kenyan government’s Community Health Strategy (30). The Kaloleni/Rabai sub-counties cover an area of approximately 909 km^2^ with a population of about 352,175 people living in approximately 47,000 households. More than half of the community depends on low-input rain-fed agriculture for household income, with high rates of poverty (70%), food insecurity (62%), and poor access to safe water (56%), latrines (80%), and refuse disposal (66%) (30).

### Data collection

Data were collected through structured household surveys administered face-to-face by trained field workers between July and December 2021. The surveys were administered in English or Kiswahili. The questionnaire captured demographic and identifying information, including age, gender, and CHU location; mental health status assessed using the Patient Health Questionnaire-9 (PHQ-9) (31); and environmental perception indicators recorded as binary variables reflecting respondents’ observations of climatic changes such as increased drought frequency, rainfall shifts, and higher temperatures. Additional modules addressed climate-related impacts including agricultural stressors (e.g., high input costs and crop pest prevalence), disruptions to water access, and details of sanitation facilities. Air pollution exposure was indirectly assessed through reported household cooking methods, lighting sources, and energy practices (32). The Swahili version of the PHQ-9 has previously been validated in Kenya, including among a community sample in Kilifi, demonstrating good reliability and construct validity and supporting its use for assessing depressive symptoms in this setting (33).

### Outcome measurement

The primary outcome was depression screening status, determined from PHQ-9 scores. PHQ-9 scores range from 0–27, with established severity categories: none/minimal (0–4), mild (5–9), moderate (10–14), moderately severe (15–19) and severe (20–27). The binary outcome for machine learning classification used a standard clinical cut-off: scores of less than 10 were classified as “No Depression,” and scores of 10 or higher as “Yes Depression” (screening positive). The PHQ-9 has been widely validated across diverse cultural and linguistic settings, including sub-Saharan Africa, with established reliability and construct validity (31).

### Predictor variables

A total of 31 variables were included, grouped into four thematic categories: environmental perception variables (binary indicators of perceived climate changes, such as shifts in rainfall patterns and prolonged drought periods); climate impact variables (nuanced responses to environmental stressors, including agricultural challenges, water scarcity, and weather-related damage); air pollution exposure proxies (derived from cooking fuel type, lighting sources, and household energy practices); and water and sanitation variables (describing primary drinking and cooking water sources and sanitation facility type) (32, 34).

### Statistical and machine learning analysis

Categorical data were presented as frequencies and percentages whereas continuous data were presented as medians and interquartile ranges (IQR). Two supervised classification algorithms were implemented: Random Forest (RF), an ensemble method aggregating multiple decision trees using bootstrap sampling for robustness to noise and modeling nonlinear relationships (28); and Extreme Gradient Boosting (XGBoost), a gradient boosting framework optimized for computational efficiency and predictive performance to capture complex predictor interactions (29). All analyses were conducted in Python using the scikit-learn library for RF and the XGBoost library for gradient boosting (35).

The dataset was split into training (80%) and independent test (20%) sets using GroupShuffleSplit by household identifier to prevent data leakage from the multimember households in the sample. This ensured that all individuals from the same household were assigned exclusively to either the training or test set, ensuring that no household appeared in both sets. Ten-fold cross-validation was performed on the training set using StratifiedGroupKFold, which maintains both class proportions and household grouping across folds. The binary outcome exhibited substantial class imbalance (14.9% positive; negative-to-positive ratio 5.7:1). For Random Forest, class_weight was set to ‘balanced’ to up-weight the minority class. For XGBoost, scale_pos_weight was set to the class ratio (5.7).

Random Forest hyperparameters were: n_estimators = 300, max_depth = 10, min_samples_split = 5, min_samples_leaf = 2, max_features = ‘sqrt’. XGBoost hyperparameters were: n_estimators = 300, max_depth = 6, learning_rate = 0.05, subsample = 0.8, colsample_bytree = 0.8, gamma = 0, reg_lambda = 1.

Model performance was evaluated on the independent test set using overall accuracy, balanced accuracy, sensitivity (recall for the positive class), specificity (recall for the negative class), precision, F1-scores for both classes, receiver operating characteristic–area under the curve (ROC-AUC), precision-recall area under the curve (PR-AUC), and Brier score. Bootstrap resampling (500 iterations) was used to estimate 95% confidence intervals for accuracy and ROC-AUC.

For RF, feature importance was determined by using the mean decrease in Gini impurity. For XGBoost, importance was calculated based on gain. Additionally, SHAP (Shapley Additive exPlanations) values were computed for both models using TreeExplainer on aa random subsample of 500 test observations to provide model-agnostic, direction-aware feature contribution (36).

Approval was obtained from the Institutional Scientific and Ethics Review Committee (ISERC) at the Aga Khan University, Nairobi (Ref: 2013/IREC-65 (v9)), and National Commission for Science Technology and Innovation (NACOSTI) (Ref: NACOSTI/P/21/11172). All recruitment procedures adhered to ethical guidelines, ensuring that participation was voluntary and participants were allowed to withdraw from the study at any time without any consequences. All participants received detailed study information in their preferred language, and written informed consent was obtained prior to data collection. Confidentiality was ensured through the removal of personal identifiable information, with all data stored in password-protected systems accessible only to the research team.

## Results

A total of 17,738 participants completed the survey, comprising 7,821 heads of households and 9,917 spouses, after excluding 2 individuals with missing age data from the original 17,740 records. The surveys were conducted across 10 Community Health Units (CHUs): Buni, Kibwabwani, Kombeni, Kwale, Mnazimwenga, Mutsengo, Mwele, Tsangatsini, Viragoni, and Vishakani. The map of the county and study locations is shown in Figure 1. The median age of participants was 41.0 years [IQR: 31.0–54.0], with 13,794 females (77.8%) and 3,944 males (22.2%). The demographic characteristics of the participants are summarized in Table 1.

**Figure 1:**
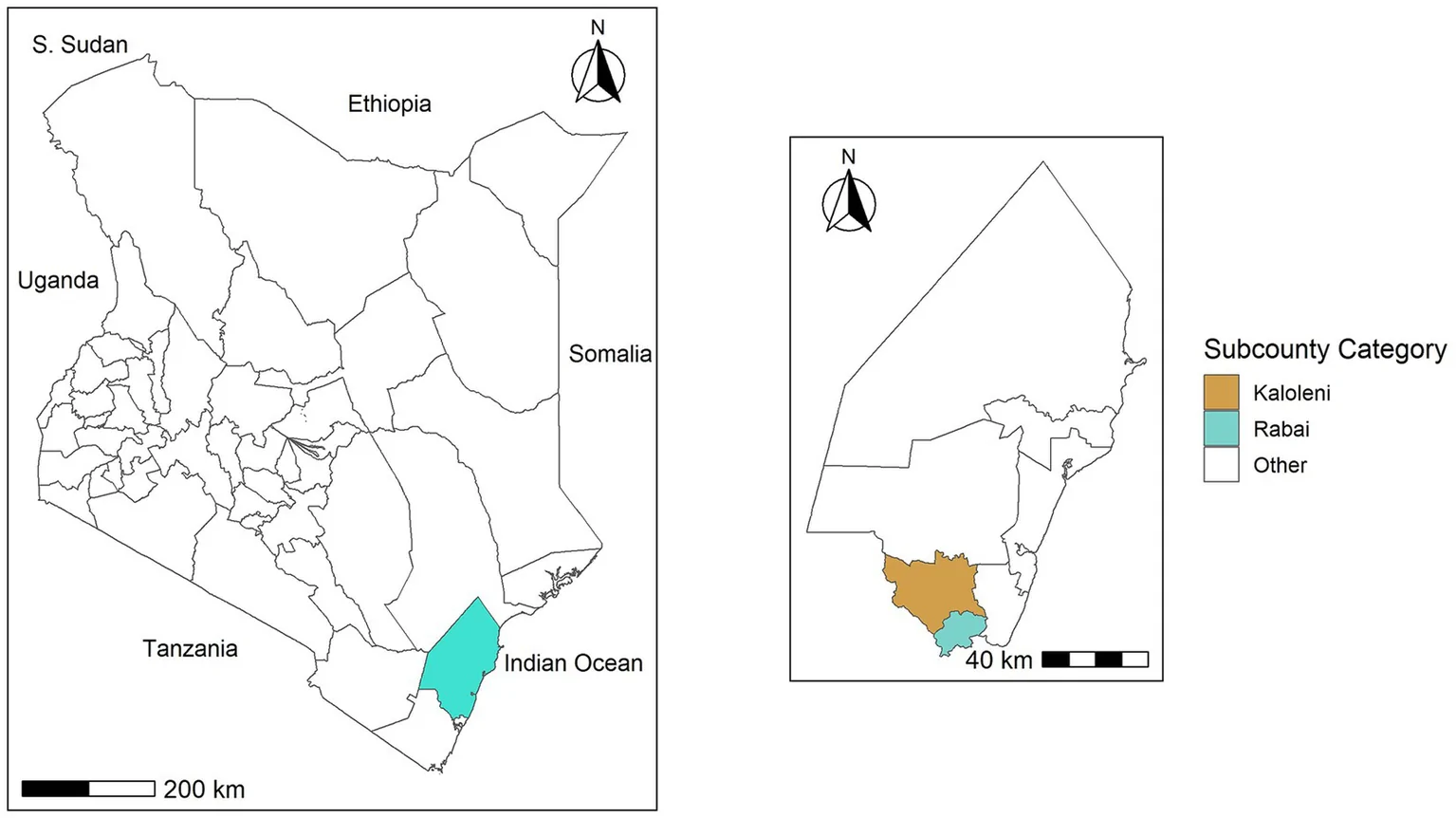
Geographic location of the 10 community health units within the Kaloleni/Rabai Health and Demographic Surveillance System, Kilifi County, Kenya.

**Table 1:** Sociodemographic characteristics of study participants.

Variable name		*n*	%
Community Health Unit	Buni	2,718	15.3%
	Kibwabwani	1,486	8.4%
	Kombeni	1,662	9.4%
	Kwale	835	4.7%
	Mnazimwenga	959	5.4%
	Mutsengo	2,215	12.5%
	Mwele	1844	10.4%
	Tsangatsini	1797	10.1%
	Viragoni	865	4.9%
	Vishakani	3,357	18.9%
Gender	Females	13,794	77.8%
	Males	3,944	22.2%
Age (median [IQR])		41.0 [31.0–54.0]	

### Depressive symptoms

The distribution of PHQ-9 severity categories were as follows: none/minimal (0–4): 47.9%, mild (5–9): 37.7%, moderate (10–14): 11.5%, moderately severe (15–19): 2.6%, severe (20–27): 0.7%. Using the standard clinical cutoff of PHQ-9 ≥ 10, 14.9% (*n* = 2,635) screened positive for depression and this binary classification was used as the outcome for machine learning modeling. Figure 2 summarizes the distribution of depressive symptoms categories.

**Figure 2:**
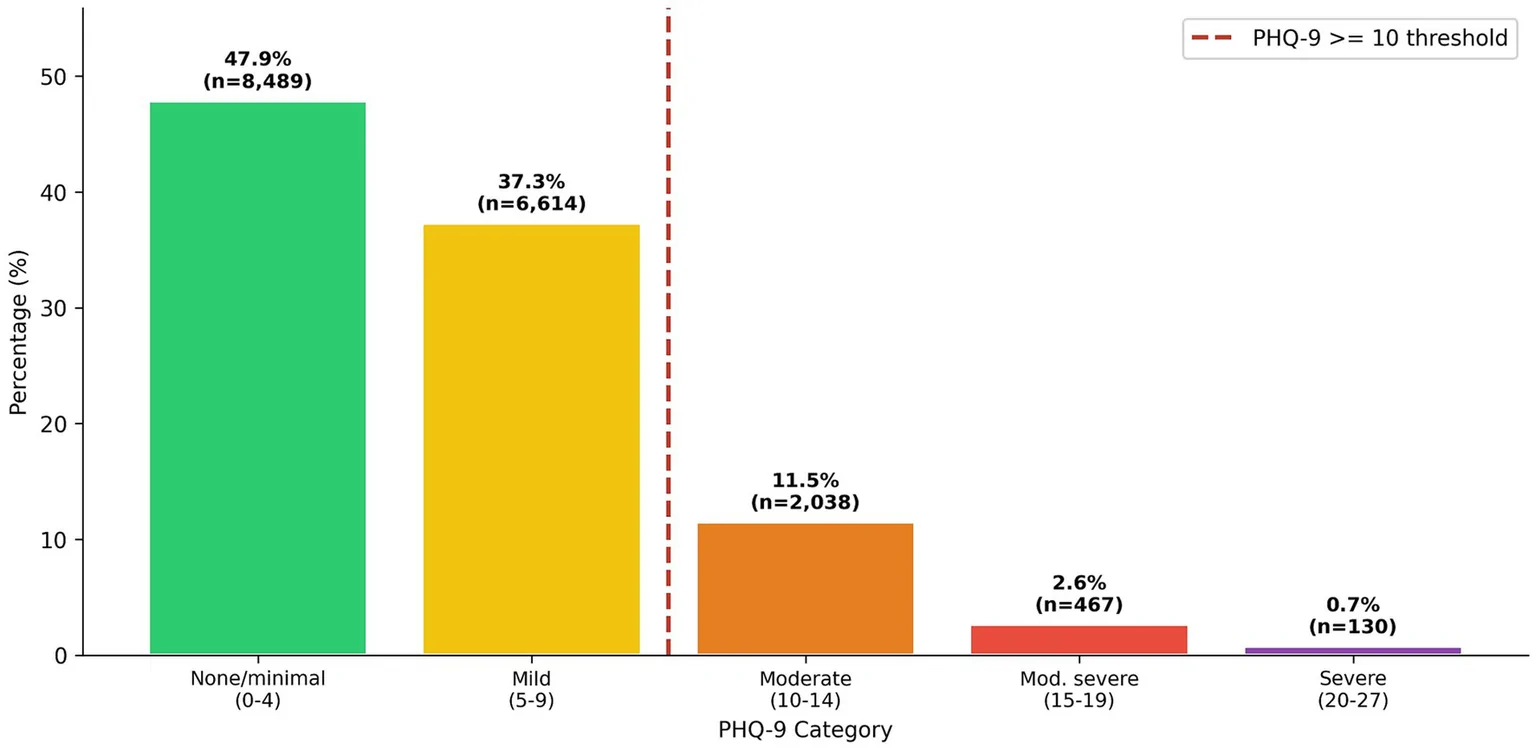
Summary of the categories of depression symptoms.

Majority of participants (82.0%, *n* = 14,545) reported having experienced significant variations in rainfall patterns (rain occurring at different times/increased rainfall/reduced rainfall). Drought (64.6%, *n* = 11,467), higher food prices (41.6%, *n* = 7,379) and irregular rains (26.6%, *n* = 4,727) were the main climatic shocks experienced by households in the past one year. More than half (64.4%, *n* = 11,422) reported being uncomfortably hot at their homes during hot seasons. Skin rash was the main observable health risk common during hot seasons (53.7%, *n* = 9,534), followed by dehydration (31.9%, *n* = 5,665).

About a third of participants (35.2%, *n* = 6,238) reported having a piped water source for drinking water and 28.9% (*n* = 5,119) reported having a piped water source for cooking and hand washing (piped water source: piped in dwelling, piped in yard/plot, piped to neighbor and public tap/standpipe). Water treatment methods included adding bleach/chlorine (40.8%, *n* = 7,235), allowing water to settle (24.1%, *n* = 4,279), boiling (25.2%, *n* = 4,471), or other methods (9.9%, *n* = 1,755). Regarding sanitation, 21.1% (*n* = 3,738) reported using pit latrines, 15.5% (*n* = 2,750) practiced open defecation, 13.7% (*n* = 2,424) used flush toilets, while other types accounted for 49.8%.

Connection to electricity was reported for 29.7% (*n* = 5,273) of the households. Majority (86.0%, *n* = 15,264) reported using open fire or stone fire (traditional stone) as cooking stove. In terms of lighting sources, open air wick lamp was commonly used (59.9%, *n* = 10,631), especially amongst residents in rural areas. A sizeable proportion of participants cooked in the rooms where they lived or slept in (44.7%, *n* = 7,933) or in a separate dwelling (42.4%, *n* = 7,530). Table 2 summarizes the climate shocks and household environmental characteristics of the study participants.

**Table 2:** Climate shocks and household characteristics of study participants.

Options	Values	*n*	%
Changes in rainfall pattern	Yes	14,545	82.0
	No	3,193	18.0
More days with very high temperatures	Yes	7,108	40.1
	No	10,630	59.9
Household affected by drought (past 12 months)	Yes	11,467	64.6
	No	6,271	35.4
Household affected by irregular rains	Yes	4,727	26.6
	No	13,011	73.4
Household affected by high food prices	Yes	7,379	41.6
	No	10,359	58.4
Home feels uncomfortably hot during summer	Yes	11,422	64.4
	No	6,316	35.6
Most critical health risk during extreme heat	Dehydration	5,665	31.9
	Skin rash	9,532	53.7
	Other	2,541	14.3
Main source of drinking water	Piped (combined)	6,238	35.2
	Tubewell/Dug well	2,407	13.6
	Surface water	4,951	27.9
	Other	4,142	23.3
Main source of water for cooking and washing	Piped (combined)	5,119	28.9
	Tubewell/Dug well	2,960	16.7
	Surface water	4,660	26.3
	Other	4,999	28.2
Type of household toilet facility	Flush (combined)	2,424	13.7
	Pit latrine (combined)	3,738	21.1
	None/Bush/Field	2,750	15.5
	Other	8,826	49.8
Method used to make water safer for drinking	Boil	4,469	25.2
	Add bleach/chlorine	7,235	40.8
	Let it stand and settle	4,279	24.1
	Other	1755	9.9
Household connected to electricity	Yes	5,273	29.7
	No	12,465	70.3
Type of cook stove used	Open fire/Stone fire	15,262	86.0
	Other types	2,476	14.0
Primary lighting source	Open wick lamp	10,629	59.9
	Other sources	7,109	40.1
Main cooking location (past 12 months)	Inside dwelling (sleeping/not sleeping area)	7,933	44.7
	Separate structure	7,530	42.5
	Other	2,275	12.8

#### Machine learning model performance

The training set contained 14.9% positive (*n* = 14,193) observations and the test set 14.6% positive (*n* = 3,545) observations, with zero household overlap between sets. Both models demonstrated moderate predictive performance with good discriminative ability. XGBoost achieved an overall accuracy of 0.802 (95% CI: 0.788–0.815), ROC-AUC of 0.845 (95% CI: 0.826–0.862), balanced accuracy of 0.753, sensitivity of 0.684, and specificity of 0.822. Random Forest achieved an accuracy of 0.796 (95% CI: 0.782–0.808), ROC-AUC of 0.823 (95% CI: 0.804–0.841), balanced accuracy of 0.738, sensitivity of 0.657, and specificity of 0.819.

For depression-positive cases (Yes Depression class), XGBoost achieved precision of 0.40, recall (sensitivity) of 0.68, and F1-score of 0.50. Random Forest achieved precision of 0.38, recall of 0.66, and F1-score of 0.48. For the No Depression class, both models achieved high precision (RF 0.93, XGB 0.94) and F1-scores (RF 0.87, XGB 0.88).

The moderate precision for the positive class (0.38–0.40) reflects the deliberate trade-off from class-imbalance handling: by increasing sensitivity from the levels that would occur without reweighting, precision is reduced. For community screening applications where identifying at-risk individuals is prioritized, this trade-off may be acceptable. Ten-fold grouped cross-validation on the training set showed consistent performance: RF mean accuracy 0.794 ± 0.009, XGBoost 0.800 ± 0.010.

The overall model performance on the independent test set is presented and summarized in Table 3. Furthermore, the confusion matrix for both models is presented in Figure 3.

**Table 3:** Model performance on the independent test set.

Metric	Random Forest	XGBoost
Overall accuracy (95% CI)	0.796 (0.782–0.808)	0.802 (0.788–0.815)
Balanced accuracy	0.738	0.753
ROC-AUC (95% CI)	0.823 (0.804–0.841)	0.845 (0.826–0.862)
PR-AUC	0.504	0.572
Brier score	0.154	0.136
Sensitivity	0.657	0.684
Specificity	0.819	0.822
Precision (yes depression)	0.38	0.40
Recall (yes depression)	0.66	0.68
F1-score (yes depression)	0.48	0.50
Precision (no depression)	0.93	0.94
Recall (no depression)	0.82	0.82
F1-score (no depression)	0.87	0.88

**Figure 3:**
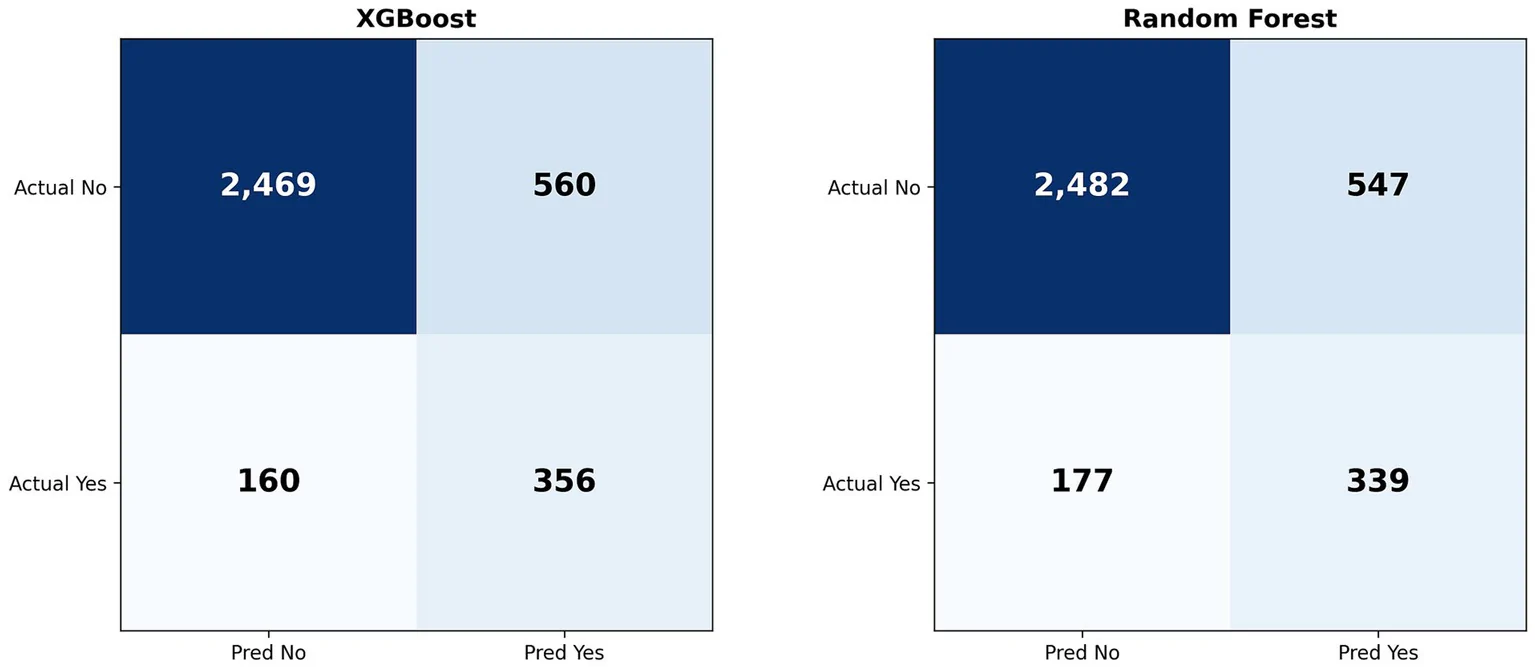
Confusion matrix of XGBoost and Random Forest.

A sensitivity analysis comparing the household-grouped split against a simple random stratified split showed minimal differences in accuracy (Δ ≤ 0.004) and modest differences in ROC-AUC (Δ ≈ 0.015), consistent with the small number of multi-member households (103/17,635). The grouped split was retained as the primary analysis as it is methodologically correct.

#### Feature importance analysis

Feature importance was assessed using three complementary methods: Gini importance (RF), gain importance (XGBoost) and SHAP values (both models). Key predictors were identified by convergence across methods, strengthening confidence in their relevance. In XGBoost, the top predictors by gain importance were open-fire cooking, drought shock, home thermal discomfort, cooking/washing water source, and perceived hotter temperatures. In RF, the top predictors by Gini importance were household drinking water source, heat-related health risk, cooking/washing water source, water treatment methods, toilet facility type and drought shock. SHAP analysis, which provides both magnitude and direction of feature contributions, confirmed these patterns. For both models, drinking water source, home thermal discomfort, drought exposure, cooking/washing water source, and heat-related health risks emerged among the most influential predictors. Cooking with open fire ranked in the top 10 by SHAP values for both models. Together, both models converge on environmental stressors as key correlates of depression screening status, with water/sanitation access, climate variability perceptions, thermal discomfort and household air pollution proxies emerging as dominant predictive factors. The feature importance is presented in Figure 4. The SHAP values are presented in Figure 5.

**Figure 4:**
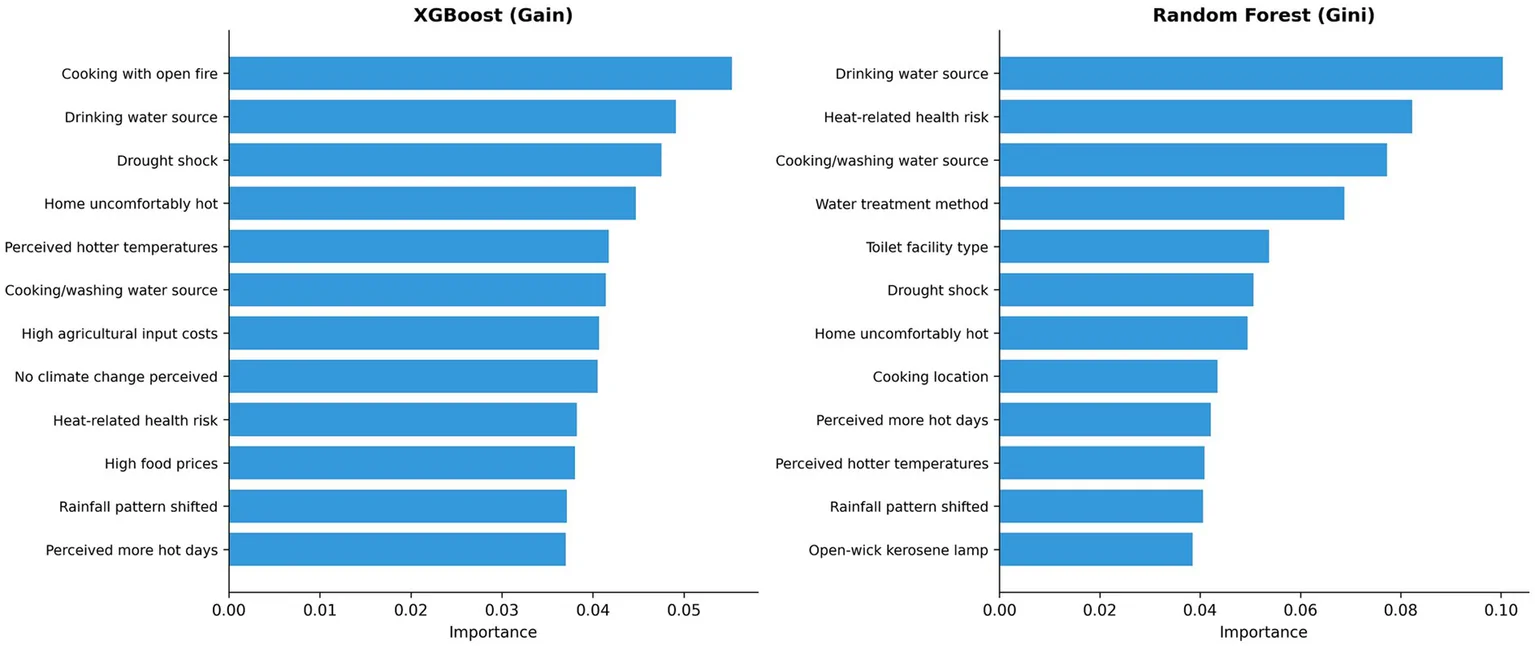
Feature importance of XGBoost and Random Forest.

**Figure 5:**
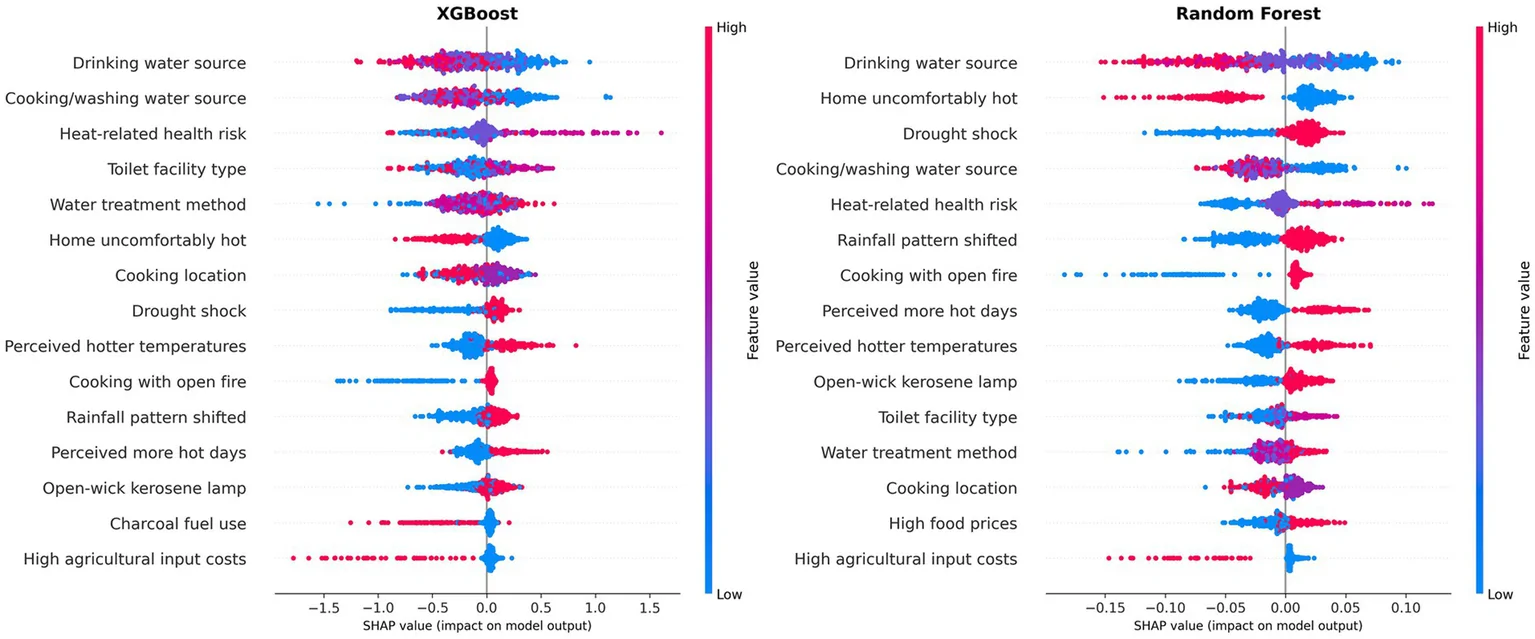
SHAP summary plots – feature contribution to depression prediction (n = 500 test observations; positive SHAP = increased predicted risk).

## Discussion

This study examined the associations between environmental and household exposures and depression screening status by applying machine learning methods to a large community-based cohort (*n* = 17,738) from Kilifi County in Kenya. The prevalence of screening positive for depression was 14.9%, while 52.1% reported having any depressive symptoms. The screening-positive prevalence is higher than national prevalence estimates for depression in Kenya (4–5% in WHO global estimates), likely reflecting symptom screening in high-adversity settings versus modeled /diagnostic sources (37). Recurrent drought, irregular rainfall, heat discomfort, and energy poverty plausibly amplify depressive symptom burden, consistent with the broader literature on poverty–mental health feedback loops and climate-related psychological risk (38). Both the XGBoost and RF models demonstrated good discriminative ability. Both the XGBoost and Random Forest models demonstrated good discriminative ability and with class-imbalance handling, both models identified approximately two-thirds of depression-positive individuals (sensitivity: XGBoost 0.684, RF 0.657), at the cost of reduced precision for the positive class (0.38–0.40). This precision-sensitivity trade-off is an important consideration for potential community screening applications: a high false-positive rate would increase the number of individuals referred for unnecessary follow-up, but the improved sensitivity ensures fewer cases of depression are missed. For settings where mental health services are scarce and initial screening can be followed by confirmatory assessment, higher sensitivity may be preferable (38). Key predictors, particularly open-fire cooking (86.0% of households) and irregular rainfall (83.1% of participants), mirror established pathways linking air pollution and climate variability to psychological distress (38, 39).

The prominence of water/sanitation variables across all three importance methods is noteworthy; inadequate water and sanitation access may contribute to psychological strain through multiple pathways including daily time burdens, safety concerns, stigma, and health risks (23). An important interpretive caveat is that many of the leading predictors — including open-fire cooking, unimproved water sources, lack of electricity, and poor sanitation — are also markers of broader socioeconomic deprivation. The models may therefore be capturing a latent poverty construct rather than isolating specific environmental exposure effects. Without income, education, and wealth indicators as covariates, this possibility cannot be resolved, and the feature importance rankings should be interpreted accordingly.

Open-fire cooking aligns with evidence associating household/indoor air pollution with depression risk in LMICs. A recent cohort-focused systematic review and meta-analysis found a significant association between indoor air pollution and depression (40). Related synthesis work on particulate matter exposure also supports associations between air pollution and common mental disorders (6), while time-series evidence links air pollution fluctuations with acute depression-related health service use (5). Air pollution may influence mood and cognitive function through neuroinflammatory and oxidative stress pathways, and its effects are compounded by poverty-related exposures that limit coping capacity, reflecting broader evidence of air pollution’s impact on health and human capital in Africa (15).

Perceived climatic variability—especially irregular rainfall and drought—also emerged as key predictors, aligning with multi-pathway models in which climate shocks undermine food security and livelihoods, thereby elevating chronic stress and the risk of depressive symptoms (41). Climate-linked psychological responses can include persistent worry, rumination, and loss-related distress; the concept of “ecological grief” further captures how environmental degradation and anticipated losses can affect mental wellbeing (42). Integrative reviews similarly conclude that climate hazards and environmental change are increasingly associated with depression, anxiety, PTSD, and related outcomes, particularly among already marginalized populations (3, 39). Food insecurity is another plausible pathway linking climate stressors to mental health, with narrative synthesis evidence in West Africa showing consistent associations between food insecurity/insufficiency and psychological distress (43). In parallel, climate-related disasters such as floods have been associated with adverse mental health outcomes in sub-Saharan Africa (44) and psychological distress in flood-prone communities (45), supporting the broader interpretation that multiple hazards, drought, flood risk, heat, and air pollution can cluster into compound mental health risks.

Thermal discomfort at home, a subjective variable reflecting both actual heat exposure and individual vulnerability, was also a leading predictor across all feature importance methods and aligns with evidence linking heat exposure to depressive symptoms through physiological stress, sleep disruption, and reduced coping capacity (2, 3). In the Kilifi context, where 64.4% of participants reported their homes being uncomfortably hot, thermal stress may represent a pervasive and under-recognized environmental contributor to psychological burden.

Methodologically, this study illustrates the utility of ML in environmental mental health research. Traditional regression models may miss nonlinearities and interactions across correlated exposures (e.g., rainfall variability co-occurring with fuel poverty and sanitation constraints). Random Forests and XGBoost are well suited to such settings because they can capture complex decision boundaries while providing feature importance signals (28, 29). Interpreting the two models together is also useful: XGBoost’s strong discrimination for the majority class can be advantageous for etiological feature ranking, while Random Forest often remains robust in the presence of correlated, noisy survey predictors.

### Strengths and limitations

This study has several strengths, including a large community-based sample, use of a validated depression screening instrument (PHQ-9), integration of multiple environmental exposure domains within an exposome-informed framework, and application of complementary machine learning methods with comprehensive evaluation metrics and SHAP-based interpretability.

Practically, the findings support integrating simple environmental risk indicators into community screening workflows, especially where routine clinical mental health services are limited. Clean cooking and household energy improvements, WASH investments, and climate-resilient livelihood support may yield mental health co-benefits alongside physical health gains. Climate–mental health policy frameworks increasingly emphasize such integrated adaptation approaches (2, 3).

Several limitations should be considered. First, the cross-sectional design prevents causal inference, and leaves open the possibility of reverse causation (e.g., depression shaping perceptions of environmental stress). Second, reliance on self-reported PHQ-9 which is a screening tool may be influenced by somatic symptom overlap and local idioms of distress, although bilingual administration likely reduced misunderstanding. Third, key potential confounders including income, education, marital status, occupation, social support, food insecurity, and prior mental health history were not available in the dataset and were not controlled for. Fourth, exposure misclassification is possible because proxies (e.g., cooking type, perceived heat discomfort) do not capture dose, duration, or household ventilation, which are central to air pollution exposure assessment. Fifth, model calibration was not formally assessed through calibration plots, and external validation on an independent sample was not performed. The generalizability of these models beyond the KRHDSS catchment area remains to be established. Sixth, common-method bias is a concern given that both the outcome (self-reported PHQ-9) and several predictors (perceived environmental conditions) were obtained from the same respondent in the same interview, which may inflate observed associations. Seventh, although the household-grouped split addressed potential data leakage from the 103 multi-member households, spatial clustering at the CHU level was not addressed through leave-one-cluster-out cross-validation, which could provide a more stringent test of geographic generalizability. Finally, the sample’s sex distribution (predominantly female) may affect generalizability to men, and future work should test model transportability across subgroups and seasons.

## Conclusion

In this large community-based cohort from rural Kilifi County, depressive symptoms were highly prevalent, highlighting a significant mental health burden in climate-stressed, low-resource settings. Machine learning models (XGBoost and Random Forest) demonstrated strong predictive performance, showing that routinely collected household and environmental indicators can enable scalable risk stratification within community surveillance systems. Key predictors—open-fire cooking, perceived heat discomfort, irregular rainfall, drought, and water and sanitation limitations—align with established pathways linking household air pollution, climatic variability, and material deprivation to psychological distress.

Although sensitivity for depression-positive cases was moderate, the approach provides practical value for identifying high-risk groups and informing targeted prevention. Future longitudinal studies incorporating refined exposure metrics (e.g., ventilation, pollutant dosimetry), richer social stressor data, and class-imbalance strategies are needed to strengthen causal inference and improve detection of depression cases.

These findings underscore the need for integrated public health interventions that combine mental health services with environmental and climate adaptation measures. Priorities include clean cooking solutions, heat mitigation, improved water and sanitation infrastructure, and climate-resilient livelihoods. Embedding these environmental risk signals into Kenya’s community health workflows could enhance early identification and guide locally tailored interventions, advancing precision public mental health in resource-constrained, climate-vulnerable settings.

## Data Availability

The raw data supporting the conclusions of this article will be made available by the authors, without undue reservation.
