# Supplementary material for: Environmental and household factors associated with depressive symptoms in rural Kilifi County, Kenya: a machine learning analysis
Source: Front Public Health. 2026 Sep 23;14:1826112. doi: 10.3389/fpubh.2026.1826112 (PMC13642830; doi:10.3389/fpubh.2026.1826112)
Supplement: Supplementary file 1 [file Table_1.docx]

| **Logistic Regression: Environmental Factors Associated with Depression (PHQ-9 ≥10), N = 17,738** | | | | | | | | | |
| --- | --- | --- | --- | --- | --- | --- | --- | --- | --- |
| *Adjusted for: Age (continuous) + Sex (Male vs Female ref) + CHU (10 categories, Vishakani ref)* | | | | | | | | | |
|  |  |  |  |  |  |  |  |  |  |
| **Variable** | **Category** | **n** | **n depressed** | **Crude OR** | **Crude 95% CI** | **Crude p-value** | **Adjusted OR** | **Adjusted 95% CI** | **Adjusted p-value** |
| **No climate change perceived** | Yes vs No | 1,176 | 208 | **1.251** | 1.071–1.463 | **0.005** | 0.874 | 0.739–1.034 | 0.117 |
| **Rainfall pattern shifted** | Yes vs No | 9,737 | 1,767 | **1.822** | 1.670–1.988 | **<0.001** | **1.277** | 1.158–1.409 | **<0.001** |
| **Perceived reduced rainfall** | Yes vs No | 7,746 | 1,113 | 0.934 | 0.859–1.015 | 0.109 | **0.795** | 0.721–0.877 | **<0.001** |
| **Perceived more rainfall** | Yes vs No | 249 | 53 | **1.561** | 1.149–2.121 | **0.004** | 1.385 | 0.990–1.938 | 0.057 |
| **Perceived hotter temperatures** | Yes vs No | 5,443 | 1,127 | **1.868** | 1.716–2.033 | **<0.001** | **1.216** | 1.074–1.378 | **0.002** |
| **Perceived more hot days** | Yes vs No | 5,006 | 1,069 | **1.936** | 1.777–2.110 | **<0.001** | 0.899 | 0.801–1.010 | 0.072 |
| **Drought shock** | Yes vs No | 11,467 | 2,018 | **1.957** | 1.778–2.154 | **<0.001** | **1.215** | 1.089–1.355 | **<0.001** |
| **Irregular rainfall shock** | Yes vs No | 4,727 | 811 | **1.270** | 1.160–1.390 | **<0.001** | **1.445** | 1.303–1.602 | **<0.001** |
| **Flood shock** | Yes vs No | 27 | 3 | 0.716 | 0.215–2.380 | 0.586 | 0.431 | 0.124–1.497 | 0.185 |
| **Landslide shock** | Yes vs No | 18 | 2 | 0.716 | 0.165–3.117 | 0.657 | 0.664 | 0.143–3.082 | 0.601 |
| **Earthquake shock** | Yes vs No | 8 | 2 | 1.911 | 0.386–9.474 | 0.428 | 3.526 | 0.514–24.171 | 0.200 |
| **High crop pest prevalence** | Yes vs No | 2,289 | 372 | **1.131** | 1.003–1.274 | **0.044** | **1.212** | 1.050–1.398 | **0.009** |
| **High livestock pest prevalence** | Yes vs No | 1,374 | 277 | **1.500** | 1.305–1.723 | **<0.001** | **1.248** | 1.066–1.463 | **0.006** |
| **Low agricultural inputs** | Yes vs No | 190 | 23 | 0.788 | 0.508–1.220 | 0.285 | **0.631** | 0.403–0.989 | **0.045** |
| **High agricultural input costs** | Yes vs No | 1,398 | 125 | **0.541** | 0.448–0.653 | **<0.001** | **0.500** | 0.409–0.611 | **<0.001** |
| **High food prices** | Yes vs No | 7,379 | 1,333 | **1.534** | 1.412–1.666 | **<0.001** | **0.794** | 0.706–0.894 | **<0.001** |
| **Home uncomfortably hot** | Yes vs No | 11,422 | 2,016 | **1.973** | 1.792–2.171 | **<0.001** | **1.460** | 1.296–1.645 | **<0.001** |
| **Household electricity** | Yes vs No | 5,273 | 735 | **0.901** | 0.821–0.987 | **0.026** | **0.792** | 0.707–0.887 | **<0.001** |
| **Cooking with open fire** | Yes vs No | 15,262 | 2,440 | **2.226** | 1.911–2.593 | **<0.001** | **1.546** | 1.309–1.826 | **<0.001** |
| **Cooking with stove** | Yes vs No | 2,287 | 219 | **0.571** | 0.494–0.661 | **<0.001** | **0.706** | 0.602–0.829 | **<0.001** |
| **Cooking with other fuel** | Yes vs No | 484 | 30 | **0.372** | 0.256–0.539 | **<0.001** | **0.275** | 0.188–0.403 | **<0.001** |
| **Charcoal fuel use** | Yes vs No | 3,032 | 367 | **0.755** | 0.671–0.850 | **<0.001** | **0.794** | 0.694–0.908 | **<0.001** |
| **Wood fuel use** | Yes vs No | 15,390 | 2,318 | **1.136** | 1.001–1.289 | **0.048** | 1.005 | 0.877–1.153 | 0.940 |
| **Candle lighting** | Yes vs No | 758 | 37 | **0.284** | 0.204–0.396 | **<0.001** | **0.277** | 0.197–0.391 | **<0.001** |
| **Open-wick kerosene lamp** | Yes vs No | 10,629 | 1,756 | **1.403** | 1.286–1.530 | **<0.001** | **1.372** | 1.247–1.509 | **<0.001** |
| **Heat-related health risk** | *Dehydration (ref)* | 5,665 | 598 | *Ref* |  |  | *Ref* |  |  |
|  | Skin rash | 9,532 | 1,471 | **1.546** | 1.397–1.711 | **<0.001** | **1.390** | 1.237–1.561 | **<0.001** |
|  | Headache/dizziness | 1,675 | 453 | **3.141** | 2.739–3.603 | **<0.001** | **1.868** | 1.574–2.217 | **<0.001** |
|  | Respiratory problems | 401 | 35 | 0.810 | 0.567–1.158 | 0.248 | 1.246 | 0.851–1.823 | 0.258 |
|  | Fatigue | 145 | 30 | **2.210** | 1.466–3.333 | **<0.001** | **1.791** | 1.169–2.742 | **0.007** |
|  | Eye irritation | 143 | 21 | 1.459 | 0.911–2.335 | 0.116 | 1.351 | 0.825–2.213 | 0.232 |
|  | Other | 177 | 27 | **1.525** | 1.004–2.318 | **0.048** | 0.890 | 0.578–1.371 | 0.597 |
| **Drinking water source** | *Other/unprotected (ref)* | 4,142 | 835 | *Ref* |  |  | *Ref* |  |  |
|  | Piped/borehole | 6,238 | 1,060 | **0.811** | 0.733–0.897 | **<0.001** | **0.817** | 0.707–0.944 | **0.006** |
|  | Rainwater | 2,407 | 263 | **0.486** | 0.419–0.564 | **<0.001** | **0.542** | 0.456–0.644 | **<0.001** |
|  | Protected spring/well | 4,951 | 477 | **0.422** | 0.374–0.477 | **<0.001** | **0.702** | 0.579–0.851 | **<0.001** |
| **Cooking/washing water source** | *Other/unprotected (ref)* | 4,999 | 965 | *Ref* |  |  | *Ref* |  |  |
|  | Piped/borehole | 5,119 | 733 | **0.699** | 0.629–0.776 | **<0.001** | **0.458** | 0.397–0.529 | **<0.001** |
|  | Rainwater | 2,960 | 427 | **0.705** | 0.622–0.798 | **<0.001** | **0.638** | 0.551–0.738 | **<0.001** |
|  | Protected spring/well | 4,660 | 510 | **0.514** | 0.458–0.577 | **<0.001** | **0.667** | 0.571–0.780 | **<0.001** |
| **Toilet facility type** | *Other (ref)* | 8,826 | 1,363 | *Ref* |  |  | *Ref* |  |  |
|  | Flush/pour-flush | 2,424 | 336 | 0.881 | 0.775–1.002 | 0.054 | 0.928 | 0.803–1.073 | 0.311 |
|  | Pit latrine | 3,738 | 534 | 0.913 | 0.819–1.017 | 0.098 | 1.030 | 0.873–1.216 | 0.724 |
|  | Open defecation | 2,750 | 402 | 0.937 | 0.831–1.058 | 0.294 | **0.805** | 0.689–0.941 | **0.006** |
| **Water treatment method** | *Other (ref)* | 1,755 | 194 | *Ref* |  |  | *Ref* |  |  |
|  | Boiling | 4,469 | 725 | **1.558** | 1.316–1.845 | **<0.001** | **1.405** | 1.168–1.689 | **<0.001** |
|  | Chlorination/chemical | 7,235 | 1,019 | **1.319** | 1.120–1.553 | **<0.001** | **1.193** | 1.003–1.419 | **0.046** |
|  | Let stand/settle | 4,279 | 697 | **1.566** | 1.321–1.856 | **<0.001** | **1.400** | 1.174–1.668 | **<0.001** |
| **Cooking location** | *Outdoors (ref)* | 2,275 | 283 | *Ref* |  |  | *Ref* |  |  |
|  | In living/sleeping room | 7,933 | 1,316 | **1.400** | 1.220–1.607 | **<0.001** | **1.408** | 1.215–1.632 | **<0.001** |
|  | Separate building | 7,530 | 1,036 | 1.123 | 0.976–1.293 | 0.106 | **1.299** | 1.119–1.509 | **<0.001** |
|  |  |  |  |  |  |  |  |  |  |
| *Bold red p-values indicate statistical significance (p < 0.05). Ref = reference category.* | | | | | | | | | |
| *climate4 recoded: original 1=Yes(hot), 2=No → analysis 1=hot, 0=not. climate13 recoded: original 1=Yes(electricity), 2=No → analysis 1=electricity, 0=not.* | | | | | | | | | |
|  |  |  |  |  |  |  |  |  |  |
| **LEGEND** |  |  |  |  |  |  |  |  |  |
| **Risk factor** | OR > 1 and p < 0.05 — higher odds of depression | | |  |  |  |  |  |  |
| **Protective** | OR < 1 and p < 0.05 — lower odds of depression | | |  |  |  |  |  |  |
| Not significant | p ≥ 0.05 — no shading; 95% CI includes 1.0 | |  |  |  |  |  |  |  |
| *Ref* | Reference category for that variable | |  |  |  |  |  |  |  |
| **p-values** | Bold red = statistically significant (p < 0.05); "<0.001" shown for very small values | | | |  |  |  |  |  |
